# Isolated skeletal muscle metastasis following successful treatment of laryngeal cancer: case report

**DOI:** 10.1186/1477-7800-7-1

**Published:** 2010-02-28

**Authors:** John R Klune, Brian Zuckerbraun, Allan Tsung

**Affiliations:** 1 Department of Surgery, University of Pittsburgh, Pittsburgh, PA, 15213, USA; 2 Department of Surgery, Veterans Affairs Pittsburgh Health System, Pittsburgh, PA, 15240, USA

## Abstract

Skeletal muscle metastases from tumors are a rare occurrence and can present difficult management decisions. We report here on a patient that had been previously treated for squamous cell laryngeal cancer with surgical resection and adjuvant systemic chemotherapy that presented with a metastasis to the rectus abdominis muscle without evidence of recurrent disease at the primary site. After a metastatic workup with PET/CT scan suggested this to be an isolated lesion, surgical excision with negative margins was performed based upon limited treatment options secondary to the location of the tumor and his favorable prognosis suggested by his pathological staging at the time of the initial resection. Here we discuss the incidence of distant metastases from laryngeal cancer and appropriate screening methods. Additionally, skeletal muscle metastases and treatment considerations are discussed.

## Introduction

Skeletal muscle metastases from cancer occur rarely and there are no clear guidelines for appropriate work-up and management of these lesions. Specifically, soft muscle metastases to the abdominal region provide exceptionally difficult treatment decisions based on location. While these can occur following percutaneous or surgical procedures in patients with head and neck cancers, it is extremely rare that one should occur in a patient without a history of any such procedures. Soft tissue metastases to muscle and skin generally represent undiscovered disease elsewhere and an overall poor prognosis. The case presented here occurred in a patient that had been successfully treated and had no signs of other recurrence on clinical exam or imaging studies. Therefore, this case presented a rare and difficult treatment strategy.

## Report of Case

**Figure 1:**
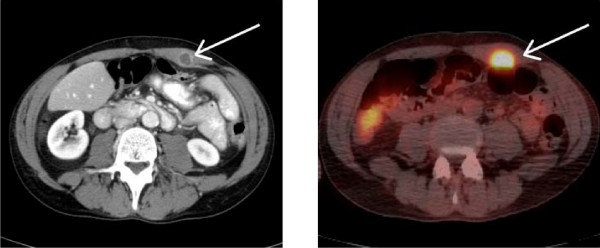
**Imaging of left rectus abdominus mass**. A.) CT scan performed during ED visit demonstrating a 14 mm lesion in the left rectus muscle. B.) Subsequent PET-CT demonstrated significant 18-FDG uptake at the site of the lesion found on CT. No other sites of abnormal uptake were found.

A 52-year-old male with a history of laryngeal cancer was found on routine physical examination to have a 2 cm subcutaneous nodule in the left mid-abdomen approximately 2 cm superior and 4 cm lateral to the umbilicus. The mass was non-mobile and slightly tender to palpation. There was no erythema, induration, or cutaneous changes. The lesion had been asymptomatic and the patient had not been aware of this finding. Computed tomography (CT) scan of the abdomen and pelvis with intravenous contrast was performed, with the only pertinent finding being the mass localized within left rectus abdominis muscle (Figure 1A). This radiographic appearance of this was interpreted as a fluid filled lesion, concerning for abscess, hematoma, or seroma. Otolaryngology and general surgery consultations were obtained.

The patient's history was significant for a squamous cell carcinoma of the larynx, treated operatively seven months prior to this presentation with a total laryngectomy and bilateral selective neck dissections of levels 2 through 4. The patient had also received adjuvant chemotherapy and local radiation. Pathology revealed a moderately to poorly differentiated squamous cell cancer of the larynx, pathologic stage T3N2bMx, with involvement of 2 out of 60 lymph nodes in the surgical specimen. The patient had been followed regularly by his primary care physician, otolaryngologist, and medical oncologist with no evidence of a local recurrence until the point of presentation. Of note, the patient did not have a history of a trans-abdominal feeding tube or any instrumentation of his abdominal wall.

Based upon the patient's above history, physical examination and CT scan appearance of the mass a fine needle biopsy was performed that revealed a moderately differentiated squamous cell carcinoma. Restaging of the patient's cancer was done by positron emission tomography/computed tomography (PET/CT) scan, which demonstrated significant fluorodeoxyglucose (FDG) uptake to the left rectus muscle with no evidence of focal uptake in the neck or elsewhere (Figure 1B). On CT, the lesion appeared to be confined to the rectus muscle within the rectus sheath, with no peritoneal or cutaneous involvement.

**Figure 2:**
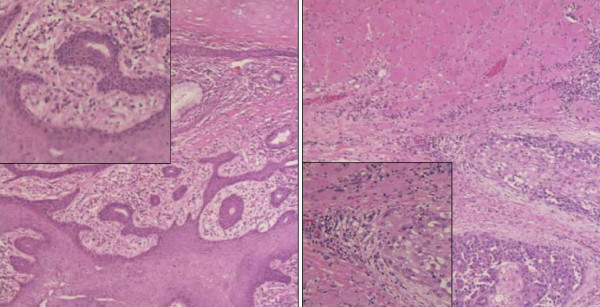
**Pathology consistent with metastatic laryngeal cancer**. A.) H & E stain histology from bilateral neck dissection demonstrating squamous cell laryngeal cancer. Corner image represents higher magnification view. B.) H & E stain histology of mass removed from the left rectus muscle, demonstrating invasive squamous cell cancer within muscle, consistent with metastatic laryngeal cancer. Corner image represents higher magnification view.

Given the finding of the isolated metastasis and the location of the mass, which limited the option of radiation treatment secondary to the high risk of radiation induced enteritis, the decision was made to proceed with surgical excision. Complete surgical excision of the lesion with minimal loss of fascia allowing for primary closure. The tumor at pathology was a 4.5 × 3.5 × 2.5 cm metastatic intramuscular squamous cell carcinoma. Histology from the original bilateral neck dissection (Figure 2A) was reviewed along with the current specimen (Figure 2B) and demonstrated that the histology from the mass was similar to that of the original laryngeal specimen. The tumor had not involved the peritoneum determined by negative margins at the posterior sheath. The tumor did involve the anterior sheath but not the skin. The patient recovered well and was discharged from the hospital. When seen in follow up, the patient was doing well and had no evidence of further disease.

## Discussion

Laryngeal cancer is the second most common type of head and neck malignancy, accounting for 25-45% of all head and neck tumors [1,2]. Squamous cell carcinoma is by far the most common histologic type, accounting for up to 90% of the cancers in this region [1]. Smoking and alcohol consumption are significant risk factors associated with approximately 85% of all laryngeal carcinomas [1,2]. Interestingly, whereas other cancer survival rates have improved significantly over the past 30 years, laryngeal cancer has demonstrated no appreciable decrease in mortality since the 1970's and it continues to be approximately 65% survival at 5 years [1].

Most commonly abdominal wall metastases for head and neck cancers, including laryngeal, occur after percutaneous, laparoscopic, or incisional procedures. Metastatic disease at the site of a percutaneous endoscopic gastrostomy (PEG) tube placement is known to occur with many different types of head and neck cancers [3-6], most widely hypothesized to be due to direct seeding by endoscopic dislodgement and displacement of cancer cells [3]. Incidence of metastatic lesions from head and neck cancers to PEG sites is unknown but estimated to be 1-3% [7,8]. It has been suggested that in these cancers, either laparoscopic [5] or radiologic [8] tube placement offers a safer alternative, although recently a case of gastrostomy-site metastasis following radiological insertion has been reported [9].

Distant metastases from head and neck cancer occur in 11-26% of patients [10,11], though one study demonstrated an incidence of distant metastases to be only 5% in patients for whom initial treatment had achieved loco-regional control [12]. The majority of these occur within 2 years [12], and the most common sites for metastases are the lung (45-85%), bones (10-30%), and liver (5-22%) [11-14]. Retrospective studies suggest that epidemiologic factors such as gender, age, or tobacco or alcohol use do not affect the incidence of distant metastases [12], but instead clinical TNM staging [15], pathologic TNM staging [12], or histologic grade of primary tumor [15] appear more closely related. The role of adjuvant radiation therapy in patients with advanced laryngeal cancer at presentation has been debated. One study demonstrated that it does not significantly affect the incidence of distant metastases but increases the risk of local recurrence and second primaries [16].

Whereas squamous cell carcinoma of the larynx has similar metastatic patterns to head and neck cancer in general, the incidence of metastases may be slightly lower [13]. Few reports of soft tissue metastases from laryngeal cancer exist, though the skin has been described as an infrequent but poor prognostic location for a metastatic lesion [13]. One case report describes metastatic laryngeal cancer to the gluteus maximus and the patient was successfully treated with surgical excision [14]. Another case report describes laryngeal cancer metastatic to the scapular muscles following treatment with total laryngectomy and bilateral neck dissection [17]. Only 3 cases can be found describing other head and neck cancers metastatic to skeletal muscles, all with varying treatments and outcomes [14]. Additionally, in a series of 500 autopsies on patients with malignant disease, 2 cases of distant metastases involving muscle are described in patients with head and neck cancers, one involving the rectus abdominus (cited in [14]).

Screening for distant metastases in head and neck cancers, and laryngeal cancer more specifically, is currently not well established. According to one study, a CT scan of the thorax is the single most important study that can be done [11]. There are few evidence based guidelines regarding imaging in soft tissue metastases after clinical detection. Some have advocated for a whole body magnetic resonance imaging (MRI) as the preferred study for evaluation [18]; however, PET/CT requires further study for its clinical benefit [19]. One recent manuscript contrasts the two modalities and concludes that PET/CT, in offering metabolic information, can be more useful in tumor localization, T-stage, and lymph node assessment [19]. In contrast, MRI can be more useful in diagnosing soft tissue metastases and differentiating metastatic disease from sarcomas and other processes [18,19]. In a recent study on imaging modalities for recurrent head and neck cancers, addition of PET/CT to traditional imaging modalities was found to significantly change the clinical management in 38.7% of patients [20]. The sensitivity and specificity of PET/CT for distant metastatic disease was 100% and 88.89% respectively [20]. Our patient received a PET/CT which demonstrated significant uptake only at the site of the abdominal wall mass, without any other identifiable lesions or lymphadenopathy. CT scan showed the lesion to be confined within the rectus sheath without any cutaneous or intraperitoneal involvement.

The treatment options for distant soft tissue metastases from all cancers are highly individualized as there is little published data. Perhaps the largest study published regarding distant soft tissue metastases included 30 new patients and a review of 91 case reports [18]. This study found that soft tissue metastases were most commonly the presenting symptom from an occult malignancy, unlike this patient that presented with a soft tissue metastasis following surgical treatment. Generally, soft tissue metastases were associated with a prognosis of 64% mortality in literature cases and 70% mortality in new patients within a mean follow-up of 7.2 and 5.4 months respectively. The authors of this case series recommend surgical excision of a soft tissue metastasis in select patients with good prognoses and solitary metastases after long disease-free intervals and appropriate treatment of the primary carcinoma. In general, they recommend chemotherapy, radiation therapy, or a combination as the preferred treatment option [18]. In the case presented above, the use of radiation as a therapeutic modality was limited given the location of the lesion with underlying small bowel that would be included in the radiation field. Additionally, this was an isolated lesion in a patient that had undergone appropriate treatment of the primary malignancy who was at relatively low cardiopulmonary risk. Of the 5 reported cases of head and neck carcinoma metastatic to skeletal muscles, 2 were treated operatively with one patient surviving disease free at 13 months of follow up while the other operative patient had died at 7 months from disease spread.

## Conclusions

This paper describes a case of a laryngeal cancer metastatic to the rectus muscle following appropriate treatment for the primary tumor. There are few described cases of distant soft tissue metastases in the literature, and therefore no evidence based guidelines exists for these occurrences. Whereas soft tissue metastases for all cancers are generally a sign of widespread disease and therefore a poor prognostic indicator, we used PET/CT to define this as an isolated lesion. The patient underwent surgical excision of the lesion with negative margins. At this point, given the low incidence of isolated soft tissue metastasis in the literature, we recommend complete whole body physical examination as an adjunct to routine follow up for head and neck cancers; however, we advocate the use of PET/CT scanning if evidence of metastatic disease is found. Treatment options need to be individualized based upon the adequacy of the initial treatment regimen, disease free interval, location of the metastasis, and the patient's cardiopulmonary risk and performance status.

## Abbreviations

PET/CT: Positron emission tomography/computed tomography; FDG: Fluorodeoxyglucose; PEG: Percutaneous endoscopic gastrostomy; MRI: Magnetic resonance imaging.

## Competing interests

The authors declare that they have no competing interests.

## Authors' contributions

RK - Manuscript design, collection of data and figures, literature review, drafting of manuscript, critical revision. BZ - Conception and manuscript design, critical revision, administrative support and supervision. AT - Conception and manuscript design, critical revision, administrative support and supervision, final approval. All authors have read and approved the final manuscript

## Consent

The authors of this report attempted to contact the patient or next of kin and were unable to contact the patient at the current contact information. He is no longer followed by the surgical clinic. Therefore, consent was unable to be obtained.

## References

[B1] Smith RV Fried MP Bailey BJ, Johnson JT Advanced cancer of the larynx Head and Neck Surgery - Otolaryngology 2006 Fourth Philadelphia, PA: Lippincott, Williams, and Williams 1757 1777

[B2] Ferlito A Shaha AR Cilver CE Rinaldo A Mondin V Incidence and sites of distant metastases from head and neck cancer J Otorhinolaryngol Relat Spec 2001 63 202 207 10.1159/000055740 11408812

[B3] Attoun A Glastonbury C Yee J Metastatic head and neck carcinoma in a percutaneous endoscopic grastrostomy site Otolaryngol Head Neck Surg 2004 131 321 323 10.1016/j.otohns.2003.09.011 15365554

[B4] Wacke W Hecker U Woenckhaus C Lerch MM Percutaneous endoscopic gastrostomy site metastasis in a patient with esophageal cancer Endoscopy 2004 36 472 10.1055/s-2004-814379 15100974

[B5] Bhama JK Haas MK Fisher WE Spread of a pharyngeal cancer to the abdominal wall after percutaneous endoscopic gastrostomy Surg Laparosc Endosc Percutan Tech 2001 11 375 378 10.1097/00129689-200112000-00008 11822863

[B6] Potochny JD Sataloff DM Spiegel JR Lieber CP Siskind B Sataloff RT Head and neck cancer implantation at the percutaneous endoscopic gastrostomy exit site Surg Endosc 1998 12 1361 1365 10.1007/s004649900858 9788864

[B7] Cruz I Mamel JJ Brady PG Cass-Garcia M Incidence of abdominal wall metastasis complicating PEG tube placement in untreated head and neck cancer Gastrointest Endosc 2005 62 708 711 10.1016/j.gie.2005.06.041 16246684

[B8] Pickhardt PJ Rohrmann CA Cossentino MJ Stomal metastases complicating percutaneous endoscopic gastrostomy: CT findings and the argument for radiologic tube placement Am J Roentgenol 2002 179 735 739 10.2214/ajr.179.3.1790735 12185055

[B9] Hawken RM Williams RW Bridger MW Lyons CB Jackson SA Puncture-site metastasis in a radiologically inserted gastrostomy tube: case report and literature review Cardiovasc Intervent Radiol 2005 28 377 380 10.1007/s00270-004-0106-5 15886946

[B10] Alvi A Johnson JT Development of distant metastasis after treatment of advanced-stage head and neck cancer Head Neck 1997 19 500 505 10.1002/(SICI)1097-0347(199709)19:6<500::AID-HED7>3.0.CO;2-2 9278758

[B11] de Bree R Deurloo EE Snow GB Leemans CR Screening for distant metastases in patients with head and neck cancer Laryngoscope 2000 110 397 401 10.1097/00005537-200003000-00012 10718426

[B12] Leon X Quer M Orus C Venegas MdP Lopez M Distant metastases in head and neck cancer patients who achieved loco-regional control Head Neck 2000 22 680 686 10.1002/1097-0347(200010)22:7<680::AID-HED7>3.0.CO;2-J 11002323

[B13] Krunic AL Cockerell CJ Truelson J Taylor RS Laryngeal squamous cell carcinoma with infradiaphragmatic presentation of skin metastases Clinical and Experimental Dermatology 2006 31 242 244 10.1111/j.1365-2230.2005.02020.x 16487102

[B14] Marioni G Blandamura S Calgaro N Ferraro SM Stramare R Staffieri A de Filippis C Distant muscular (gluteus maximus muscle) metastasis from laryngeal squamous cell carcinoma Acta-Otolaryngologica 2005 125 678 682 10.1080/00016480410024613 16076722

[B15] Matsuo JMS Patel SG Singh B Wong RJ Boyle JO Kraus DH Shaha AR Zelefsky MJ Pfister DG Shah JP Clinical nodal stage is an independently significant predictor of distant failure in patients with squamous cell carcinoma of the larynx Ann Surg 2003 238 412 422 14501507 10.1097/01.sla.0000086660.35809.8a PMC1422706

[B16] Yilmaz T Hosal S Ozyar E Akyol F Gursel B Post-operative radiotherapy in advanced laryngeal cancer: Effect on local and regional recurrence, distant metastases and second primaries Journal of Laryngology and Otology 2005 119 784 790 16259655 10.1258/002221505774481183

[B17] Yucel EA Demirel T Demiryont M Egeli U Deger K An unusual metastatic site of laryngeal carcinoma: scapular muscles Journal of Laryngology and Otology 2003 117 85 87 12590867 10.1258/002221503321046757

[B18] Damron TA Heiner J Distant soft tissue metastases: A series of 30 new patients and 91 cases from the literature Annals of Surgical Oncology 2000 7 526 534 10.1007/s10434-000-0526-7 10947022

[B19] Schmidt GP Kramer H Reiser MF Glaser C Whole-body magnetic resonance imaging and positron emission tomography-computed tomography in oncology Top Magn Reson Imaging 2007 18 193 202 10.1097/RMR.0b013e318093e6bo 17762383

[B20] Pantvaidya GH Agarwal JP Deshpande MS Rangarajan V Singh V Kakade A D'Cruz AK PET-CT in recurrent head neck cancers: A study to evaluate impact on patient management J Surg Oncol 2009 100 401 403 10.1002/jso.21257 19235784

